# Identification and Analysis of Biomarkers Associated with Lipophagy and Therapeutic Agents for COVID-19

**DOI:** 10.3390/v16060923

**Published:** 2024-06-07

**Authors:** Yujia Wu, Zhenlin Wu, Qiying Jin, Jinyuan Liu, Peiping Xu

**Affiliations:** 1 Institute of Tropical Medicine, Guangzhou University of Chinese Medicine, Guangzhou 510405, China; 20221111001@stu.gzucm.edu.cn (Y.W.); 20221111000@stu.gzucm.edu.cn (Z.W.); kimky1225@163.com (Q.J.); 2 Basic Medical College, Guangzhou University of Chinese Medicine, Guangzhou 510405, China; cszxb@gzucm.edu.cn

**Keywords:** bioinformatics, lipophagy-related genes, COVID-19, lipid metabolism, therapeutic agents

## Abstract

Background: Lipids, as a fundamental cell component, play an regulating role in controlling the different cellular biological processes involved in viral infections. A notable feature of coronavirus disease 2019 (COVID-19) is impaired lipid metabolism. The function of lipophagy-related genes in COVID-19 is unknown. The present study aimed to investigate biomarkers and drug targets associated with lipophagy and lipophagy-based therapeutic agents for COVID-19 through bioinformatics analysis. Methods: Lipophagy-related biomarkers for COVID-19 were identified using machine learning algorithms such as random forest, Support Vector Machine-Recursive Feature Elimination, Generalized Linear Model, and Extreme Gradient Boosting in three COVID-19-associated GEO datasets: scRNA-seq (GSE145926) and bulk RNA-seq (GSE183533 and GSE190496). The cMAP database was searched for potential COVID-19 medications. Results: The lipophagy pathway was downregulated, and the lipid droplet formation pathway was upregulated, resulting in impaired lipid metabolism. Seven lipophagy-related genes, including *ACADVL*, *HYOU1*, *DAP*, *AUP1*, *PRXAB2*, *LSS*, and *PLIN2*, were used as biomarkers and drug targets for COVID-19. Moreover, lipophagy may play a role in COVID-19 pathogenesis. As prospective drugs for treating COVID-19, seven potential downregulators (phenoxybenzamine, helveticoside, lanatoside C, geldanamycin, loperamide, pioglitazone, and trichostatin A) were discovered. These medication candidates showed remarkable binding energies against the seven biomarkers. Conclusions: The lipophagy-related genes *ACADVL*, *HYOU1*, *DAP*, *AUP1*, *PRXAB2*, *LSS*, and *PLIN2* can be used as biomarkers and drug targets for COVID-19. Seven potential downregulators of these seven biomarkers may have therapeutic effects for treating COVID-19.

## 1. Introduction

The outbreak of coronavirus disease 2019 (COVID-19), a highly transmissible and fatal respiratory illness caused by severe acute respiratory syndrome coronavirus 2 (SARS-CoV-2), has significantly harmed public health globally [1]. The SARS-CoV-2 virus continues to mutate and undergo genetic recombination, and there is a lack of complete understanding of the virus and its mechanism of infection. Currently, there are no specific drugs available for treating COVID-19 infection and its associated pneumonia. Clinical treatment of COVID-19 often involves the use of antiviral drugs such as remdesivir and ritonavir, together with immunosuppression and hormonal therapy, to alleviate patients’ symptoms [2].

Lipids are a crucial cellular component that play a critical role in regulating various biological processes in cells and in facilitating viral infection [3]. Viruses and typical intracellular parasites depend on lipids at every stage of infection and manipulate the lipid metabolism pathways of host cells to create a favorable environment for their own replication [4]. Lipophagy, an intracellular pathway for fat degradation, involves the transportation of intracellular lipid droplets and cholesterol as cargo through autophagosomes to lysosomes. These lipid droplets are then degraded into glycerol and free fatty acids by acid lipase and hydrolase after fusion [5]. During lipophagy, triglycerides in lipid droplets are hydrolyzed into free fatty acids, which subsequently induce mitochondrial β-oxidation to generate adenosine triphosphate (ATP) for cells. Lipophagy not only helps maintain the balance of intracellular lipid metabolism but also creates favorable conditions for the infection and replication of pathogenic microorganisms [6].

Lipophagy plays a crucial role in both the phagocytosis of pathogens by immune cells and as a cell survival mechanism for protecting cells against different stresses and injuries and maintaining tissue homeostasis [7]. In patients who died due to COVID-19, double membrane vesicles (DMVs) filled with viral RNA were detected in lung tissues with lipid droplets (LDs) located adjacent to the DMVs [8]. Several pathogenic microorganisms, including SARS-CoV-2, hepatitis B virus (HBV), hepatitis C virus (HCV), and dengue virus, inhibit lipophagy and weaken immune responses [9]. In obese individuals, the excessive accumulation of lipid droplets in various organs can accelerate the replication of SARS-CoV-2 replication and hinder its elimination through mechanisms related to lipid overload [10]. Therefore, the regulation of lipophagy has potential for diagnostic and therapeutic applications in COVID-19. To date, no study has investigated this specific process; the present study is the first to analyze the association between lipophagy and COVID-19.

The present study aimed to identify biomarkers and drug targets of lipophagy during COVID-19 and discover therapeutic agents for COVID-19. For this purpose, we constructed tissue-specific clusters to predict immune cell composition and examined the correlation between lipophagy regulators and the immune cell infiltration landscape. We utilized the COVID-19 scRNA-seq (GSE145926) and bulk RNA-seq (GSE183533 and GSE190496) datasets from the Gene Expression Omnibus (GEO) database. The identified biomarkers were further validated using external datasets. Additionally, we also explored COVID-19-targeted small-molecule drugs based on these biomarkers. The present study provides a comprehensive understanding of the molecular pathogenesis of COVID-19 and identifies valuable biomarkers and drug targets for treating COVID-19.

## 2. Methods

### 2.1. Data Download

The GSE145926, GSE183533, and GSE190496 microarray data were downloaded from the GEO database (http://www.ncbi.nlm.nih.gov/geo/, accessed on 12 March 2023). The COVID-19 scRNA-seq data GSE145926 included bronchoalveolar lavage fluid (BALF) from 6 severe and 3 moderate COVID-19 patients and 3 healthy controls, 23,742 genes, and 67,469 cells. The GSE183533 comprised 31 dead COVID-19 patients and 10 healthy non-COVID-19 individuals and 58,040 genes, and this dataset was deemed to be the discovery set for the principal analysis of this research. The GSE190496 comprised 5 normal and 8 COVID-19 FFPE BALF, and 17,883 genes were applied as the validation set.

### 2.2. scRNA-Seq Data Analysis

The single-cell transcriptome dataset was analyzed using the Seurat framework developed as an R package for clustering and representation purposes. For cluster definition, we utilized the graph-based clustering approach implemented in Seurat, and for the visual representation of the cells, t-SNE dimensionality reduction was employed. Uniform manifold approximation and projection (UMAP) were applied to explore the scRNA-seq data. The SingleR package (v0.2.1) [11], CellMarker dataset, and previous studies about lipophagy were applied to recognize the different cell types and pathways [12].

### 2.3. Identification of Differentially Expressed Genes (DEGs)

DEGs between COVID-19 patients and the control group derived from the scRNA cells and RNAseq were identified using the DEG analysis in Seurat and the edgeR package in R, respectively. The statistical threshold for significance was set at a false discovery rate (FDR) < 0.05.

### 2.4. Identification and Functional Enrichment Analysis of Lipophagy-Related DEGs in Lung Tissue of COVID-19 Patients

The dataset was normalized by group using the R packages ‘sva’ and ‘limma’ to analyze differential expression for RNA sequencing and microarray studies. Lipophagy-related DEGs were analyzed using gene ontology (GO) and the Kyoto Encyclopedia of Genes and Genomes (KEGG), as described above [13].

### 2.5. Identification of Lipophagy-Related Biomarkers

Lipophagy-related DEGs were used to identify significant biomarkers for diagnosing COVID-19. The GSE183533 dataset was utilized to build random forest (RF), support vector machine (SVM), XGBoost (extreme gradient boosting), and generalized linear model (GLM) models. The ‘DALEX’ R package’s explain function was employed to analyze these models, plot the distribution of residuals, and select the best model based on the discovery set. The significance of each variable was assessed, and the 10 most important explanatory variables were chosen for further investigation [14]. A nomogram model for predicting the occurrence of COVID-19 was developed using the ‘root mean square’ method. The ‘Score’ represents the scores of the factors mentioned below, and the ‘total score’ is the sum of the scores of these factors. Calibration curves were then used to evaluate the predictive power of the nomogram model. Finally, the clinical value of the model was assessed using decision curve analysis and a clinical response curve.

The top 10 important genes were identified using DEG and receiver operating characteristic (ROC) analysis as the most significant biomarkers from these four algorithms. The validation set was used to validate the gene expression differences for 40 major genes. The ‘pROC’ R package was used to evaluate the dominant genes in the discovery and validation sets. The predictive reliability of the biomarkers was assessed using the ROC curve, and the area under the curve (AUC) was obtained. Additionally, a logistic regression signature with these biomarkers was established to assess diagnostic ability, and the ROC curve was used to present the results.

### 2.6. Functional Enrichment Analyses

To examine the differential activities of pathways in cells derived from COVID-19 or control tissue, we performed gene set variation analysis (GSVA) using the GSEABase package [15]. We utilized a curated database to evaluate the activities of immune, cell death, and lipid metabolic pathways. The GSVA package was employed to assign pathway activity scores to each cell type. In order to investigate the potential molecular mechanisms of the immune- and lipid-related genes, we obtained comprehensive gene sets related to immune, cell death, and lipid pathways from the MSigDB database (https://www.gsea-msigdb.org/gsea/msigdb/index.jsp, accessed on 12 March 2023). These gene sets were then used to identify differentially expressed immune and lipid genes between the COVID-19 cell and alveolar cell clusters. Spearman’s coefficient was utilized to assess the correlation between cells, COVID-19, and the immune, cell death, and lipid metabolic pathways.

### 2.7. Immune Cell Infiltration Analysis

Single-sample gene set enrichment analysis (ssGSEA) was conducted using the GSVA package (v1.18.0) to estimate the immune cell score. The gene set related to immune cells was obtained from the SingleR package (v0.2.1) and the CellMarker dataset. Violin plots were used to illustrate the expression differences of the immune-infiltrating cells. Spearman correlation analysis was performed to investigate the associations between different immune-infiltrating cells. Additionally, a similar method was employed to explore the correlation between lipophagy-related biomarkers and immune cells. These results were visualized using the ‘ggplot2’ package. A *p*-value of less than 0.05 was considered statistically significant.

### 2.8. Drug Prediction Targeting Biomarkers

The cMAP (Broad Institutes) database (https://www.broadinstitute.org/connectivity-map-cmap, accessed on 13 March 2023) was used to predict the possible key gene-interacting molecule compounds. In this study, cMAP was applied to identify gene-targeted drugs, and the DrugBank database (https://www.drugbank.ca/, accessed on 13 March 2023) was used to identify the drugs’ structural elements. Small molecules or drugs with high absolute enrichment values may downregulate the gene expression of biomarkers, potentially providing therapeutic effects for COVID-19. Detailed information about the potential therapeutic agents was obtained from PubChem (https://pubchem.ncbi.nlm.nih.gov/, accessed on 16 March 2023).

### 2.9. Molecular Docking Analysis

Seven lipophagy feature proteins were considered as potential drug targets. The molecular docking simulations were carried out using the method of AutoDock Vina [16]. Protein crystal structures, including *LSS* (PDB: 1W6K), *ACADVL* (PDB: 3B96), *PRKAB2* (PDB: 6b2e), *AUP1* (PDB: 7LEW), *DAP* (Uniprot: AF-P51397-F1-model_v4), *HYOU1* (Uniprot: AF-Q9Y4L1-F1-model_v4), and *PLIN2* (Uniprot: AF-Q99541-F1-model_v4), were obtained from PDB database (https://www.rcsb.org/pdb, accessed on 14 March 2023) and Uniprot database (https://www.uniprot.org/, accessed on 13 March 2023) in pdb format. The structures of 10 drugs predicted to downregulate 7 biomarkers from the cMAP database were obtained from the PubChem database (https://pubchem.ncbi.nlm.nih.gov/, accessed on 16 March 2023) in sdf format. Proteins and drugs were prepared using AutoDockTools (v1.5.6). The molecular graphics were prepared using PyMOL (v2.3). Each molecule was supplemented with hydrogen and Gasteiger charges. The docking areas and AutoGrid parameters were determined based on the binding pockets of the proteins. The employed Ligplot + v2.2 [17] was used to analyze the interaction between protein and ligand.

### 2.10. Statistical Analysis

All bioinformatics analyses were performed using R4.0.3 or Perl software (v5.32.1.1). ROC curves were drawn, and graphs were merged using GraphPad Prism 5.0 (GraphPad Software Inc., San Diego, CA, USA). Statistical analysis was performed using version 18.0 (SPSS, Chicago, IL, USA). Analysis of variance (ANOVA) was used to compare multiple gene expressions in COVID-19 patients with different severities; *p* < 0.05 means the difference is statistically significant.

## 3. Results

### 3.1. Clustering and Cell Type of scRNA-Seq Data

**Figure 1:**
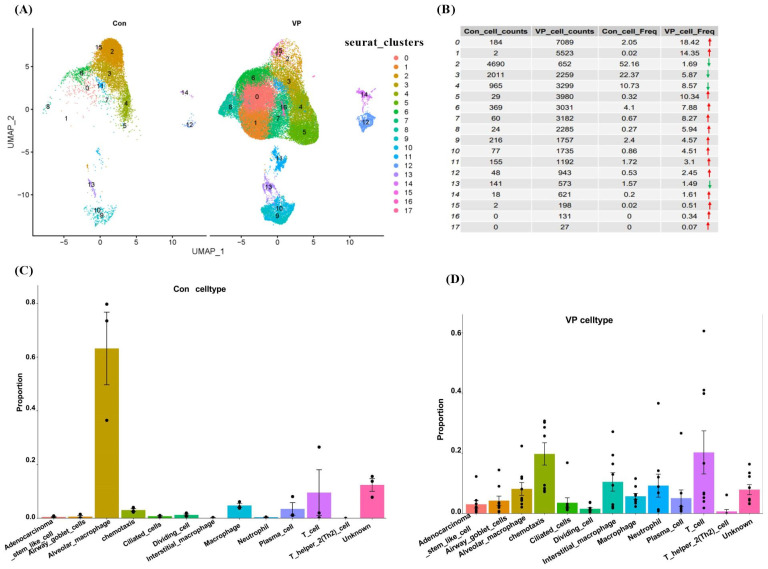
Overview of cluster and cell type of scRNA-Seq data. (**A**) Label colors according to separate clusters. (**B**) Cell counts and frequencies in the Con and VP groups. (**C**) Plot of cell type proportion in the Con group. (**D**) Plot of cell type proportion in the VP group. Con: control group; VP: viral pneumonia of the COVID-19 group.

The scRNA-seq dataset GSE145926 was utilized to analyze the heterogeneity of COVID-19 (Figure 1). Nonlinear dimension reduction using UMAP was performed, resulting in the clustering of cells into 17 clusters (Figure 1A). The frequencies of cells in clusters 0–1, 5–12, and 14–17 were higher in the VP group compared to the Con group (Figure 1B). Furthermore, the VP group exhibited higher proportions of adenocarcinoma stem-like cells, airway goblet cells, chemotaxis cells, ciliated cells, interstitial macrophages, macrophages, neutrophils, plasma cells, and T cells compared to the Con group (Figure 1C,D). However, the proportion of alveolar macrophages was lower in the VP group than in the Con group (Figure 1C,D).

### 3.2. Identification of Lipophagy-Related DEGs

**Figure 2:**
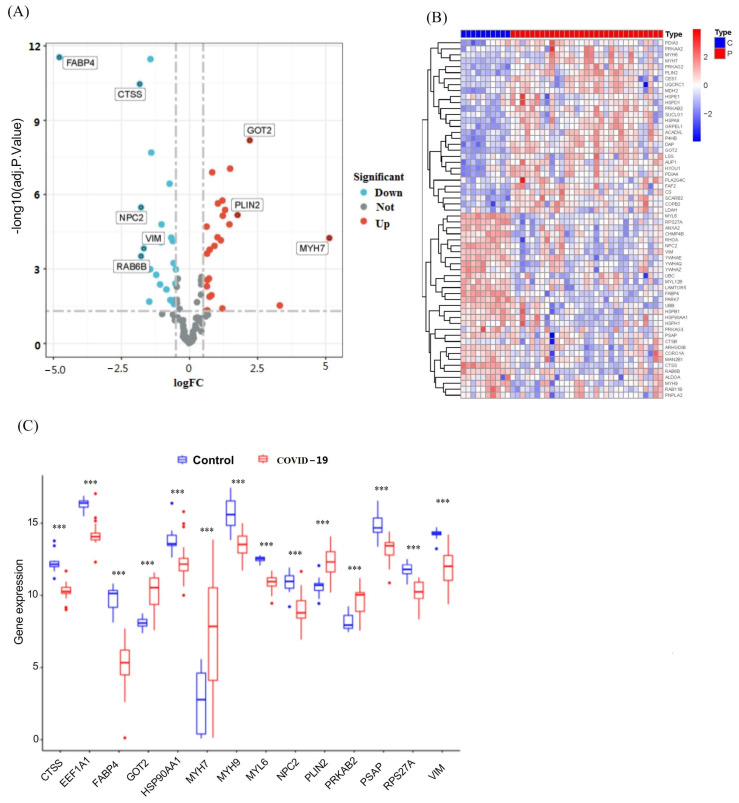
Identification of lipophagy-related DEGs in COVID-19 patients. (**A**) Volcano map showing the expression characteristics of lipophagy-related DEGs. (**B**) Heat map of 47 upregulated and downregulated genes’ DEGs. (**C**) Difference in expression of lipophagy-related genes (with the criterion of FDR < 0.05 and|log_2_(FC)| > 1.5) in COVID-19 (red) and control (blue) tissue. Blue dots represent control samples, and pink dots represent COVID-19 samples. Red or sapphire blue dots represent genes that were significantly upregulated or downregulated, respectively. The X axis represents the corrected *p*-value (scale conversion using logarithm), and the Y axis represents the logFC. Each dot in the figure represents a gene; red or cyan dots represent genes that were significantly upregulated or downregulated, respectively, and gray dots represent genes that have no difference in expression between control samples and COVID-19 samples. ***, *p* < 0.001, COVID-19 group compared to control group.

Based on the GSE183533 dataset, lipophagy-related DEGs were identified between the COVID-19 and control samples. Genes associated with lipophagy-related DEGs were selected by differential analysis between control and influenza tissues (adj. *p* < 0.05 and log2 FC > 0.5) (Figure 2). After conducting a combined analysis of lipophagy-related genes and DEGs of COVID-19, 47 genes were screened out as lipophagy-related DEGs in COVID-19, including 27 downregulated genes and 20 upregulated genes. Heat maps and volcano maps show the distribution of expressed genes between influenza and control tissues (Figure 2A). Detailed information on these DEGs is presented in Table 1. Among them, the expression levels of *ACADVL*, *AUP1*, *COPB2*, *CS*, *DAP*, *GOT2*, *GRPEL1*, *HSPA9*, *HYOU1*, *LSS*, *MDH2*, *MYH7*, *P4HB*, *PDIA3*, *PDIA4*, *PLIN2*, *PRKAB2*, *PRKAG2*, *SCARB2*, and *SUCLG1* were upregulated, whereas *ANXA2*, *ARHGDIB*, *CHMP4B*, *CORO1A*, *CTSS*, *FABP4*, *HSP90AA1*, *HSPB1*, *LAMTOR5*, *LDAH*, *MAN2B1*, *MYH9*, *MYL12B*, *MYL6*, *NPC2*, *PARK7*, *PSAP*, *RAB11B*, *RAB6B*, *RHOA*, *RPS27A*, *UBB*, *UBC*, *VIM*, *YWHAE*, *YWHAQ*, and *YWHAZ* were downregulated in COVID-19 samples than those in non-COVID-19 controls (Figure 2B). The difference gene expressions in logFC ≥ 1.5 between the COVID-19 and control samples were *CTSS*, *EEF1A1*, *FABP4*, *GOT2*, *HSP90AA1*, *MYH7*, *MYH9*, *MYL6*, *NPC2*, *PLIN2*, *PRKAB2*, *PSAP*, *RPS27A*, and *VIM* (Figure 2C).

### 3.3. Enrichment Analyses for the Lipophagy-Related DEGs

**Figure 3:**
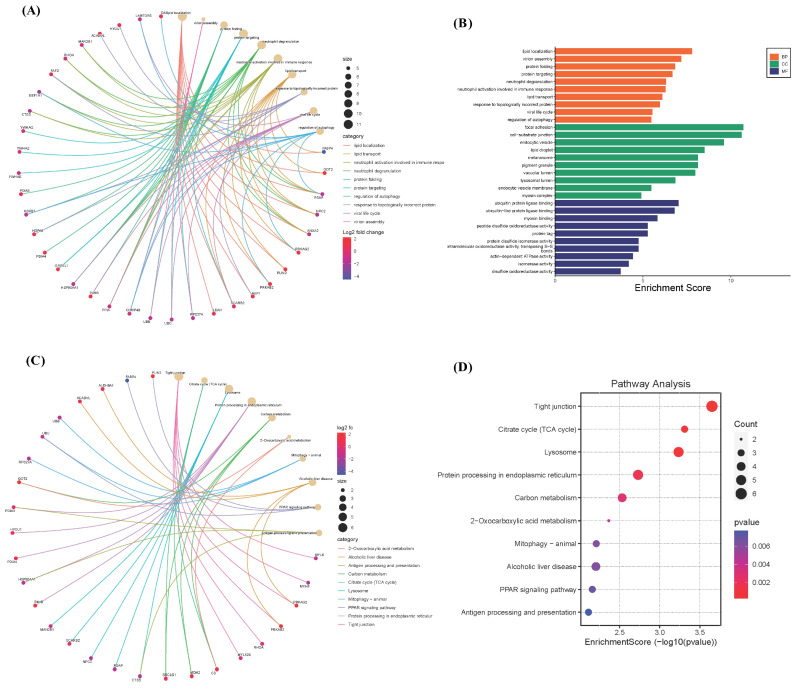
Functional enrichment analysis of lipophagy-related DEGs. (**A**) Cnetplot of BP; (**B**) GO analysis. GO enrichment analysis is presented in three parts: biological process (BP), cellular component (CC), and molecular function (MF). Four crucial mitochondria-related cellular components are marked by the purple box; (**C**) Cnetplot of KEGG pathway; (**D**) KEGG pathway enrichment analysis shows the primary pathways enriched by the 47 lipophagy-related DEGs.

We conducted GO and KEGG enrichment analysis on DEGs related to lipophagy, aiming to uncover the potential molecular biological characteristics of COVID-19. The GO enrichment analysis revealed that these genes were primarily involved in the regulation of lipids (Figure 3A,B). Additionally, the KEGG pathway analysis demonstrated that these DEGs were associated with positive regulation of the citrate cycle (TCA cycle), tight junctions, lysosomes, and carbon metabolism (Figure 3C,D). Notably, lipophagy-related DEGs were also significantly enriched in processes such as lipid localization, lipid transport, lipid droplet formation, and lipid-related signatures, including the regulation of autophagy, energy metabolism, and oxidoreductase activity. These findings provide evidence that lipophagy-related DEGs may play a crucial role in the development of COVID-19 by modulating lipid metabolism and cell death processes.

### 3.4. Identification of Lipophagy-Related Diagnostic Biomarkers for COVID-19

**Figure 4:**
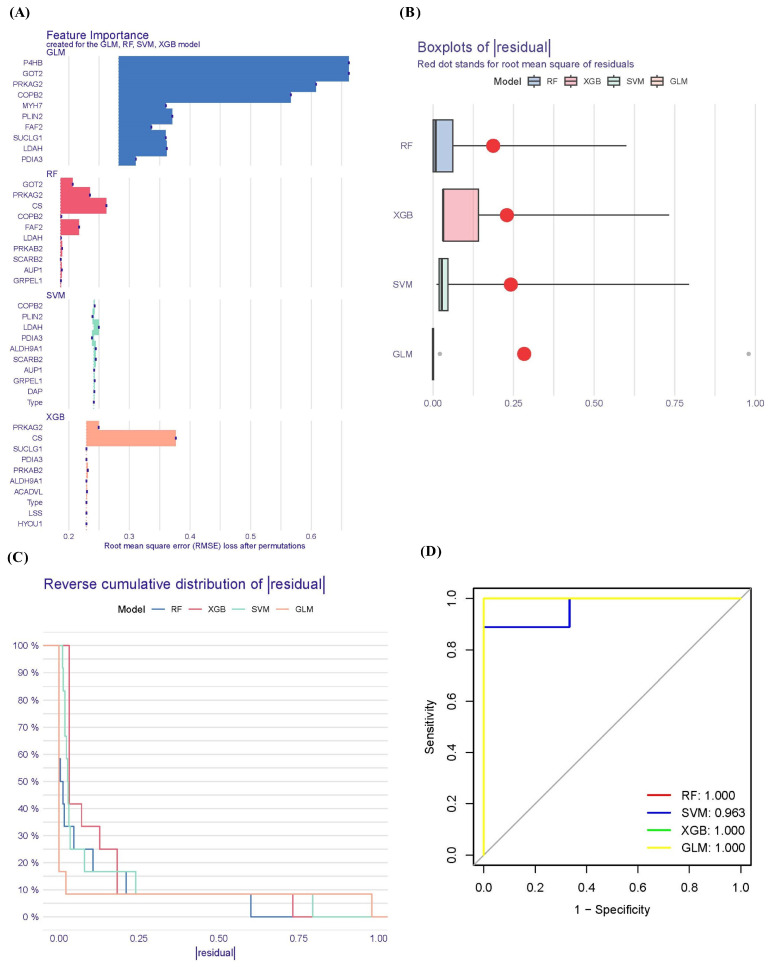
Identification of lipophagy-related diagnostic genes for COVID-19. (**A**) The importance of the variables according to root mean square error (RMSE) loss after permutations in the RF, XGB, GLM, and SVM models. (**B**) Boxplots of the residuals of the sample. Red dot stands for the root mean square of residuals. (**C**) Cumulative residual distribution map of the sample. (**D**) ROC curves for the RF, XGB, GLM, and SVM models.

To explore the potential pathogenesis of COVID-19, we conducted a comprehensive evaluation of the diagnostic values of lipophagy-related DEGs. We utilized the ‘DALEX’ package in R, which includes machine learning algorithms such as RF, XGB, GLM, and SVM. These algorithms were employed to identify the top 20 upregulated lipophagy-related DEGs, enabling the differentiation of COVID-19 from the control samples. Independently, RF, SVM, XGB, and GLM models were created using the training GSE183533 dataset. Figure 4 illustrates the performance of these four machine learning models, demonstrating their superior sample residual values (Figure 4A,C) and AUC values greater than 0.9 (Figure 4D). Among these models, RF, XGB, and GLM displayed the highest performance, exhibiting minimal sample residual and an AUC value of 1.000 (Figure 4D). Subsequently, we selected 40 top important genes from these 4 machine learning models for further analysis (Figure 4A).

**Figure 5:**
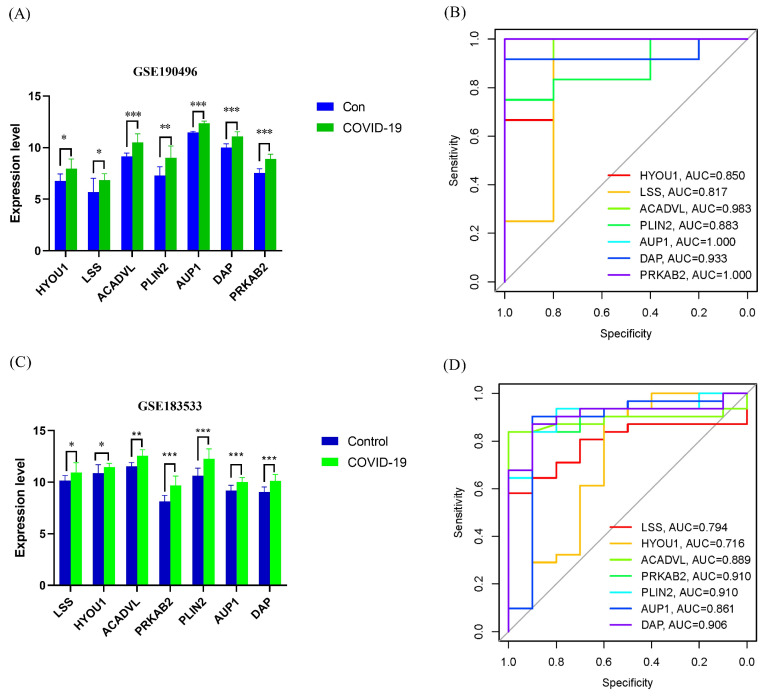
Validation of the diagnostic values of the lipophagy-related biomarkers. (**A**) Differential expression of seven lipophagy-related biomarkers between the IVP group and control group using the Wilcoxon test method in the validation set (GSE190496). (**B**) ROC curves for seven lipophagy-related biomarkers in the validation set. (**C**) Differential expression of seven lipophagy-related biomarkers between the IVP group and control group using the Wilcoxon test method in the discovery set (GSE183533). (**D**) ROC curves for seven lipophagy-related biomarkers in the discovery set. Data represent the mean ± SD. * *p* < 0.05, ** *p* < 0.01, *** *p* < 0.001 COVID-19 vs. control.

In the validation set (GSE190496), we observed a significant increase in the gene expression levels of *LSS*, *HYOU1*, *ACADVL*, *PRKAB2*, *PLIN2*, *AUP1*, and *DAP* in the COVID-19 group compared to the control group (Figure 5A). These findings are consistent with the discovery set (Figure 5C). To assess the diagnostic accuracy of these genes in distinguishing COVID-19 from non-COVID-19 controls, we plotted the ROC curves for the seven genes. As shown in Figure 5B,D, the AUCs for all seven genes were above 0.7, indicating their good diagnostic performance. In the discovery set, the AUC values for the seven genes ranged from 0.716 to 0.910 (Figure 5D). Similarly, in the validation set, the AUC values for all seven genes were above 0.8, ranging from 0.817 to 1.000 (Figure 5B). These results provide strong evidence that the seven feature genes can be considered valuable diagnostic markers for COVID-19.

### 3.5. Immunological Infiltration Analysis

**Figure 6:**
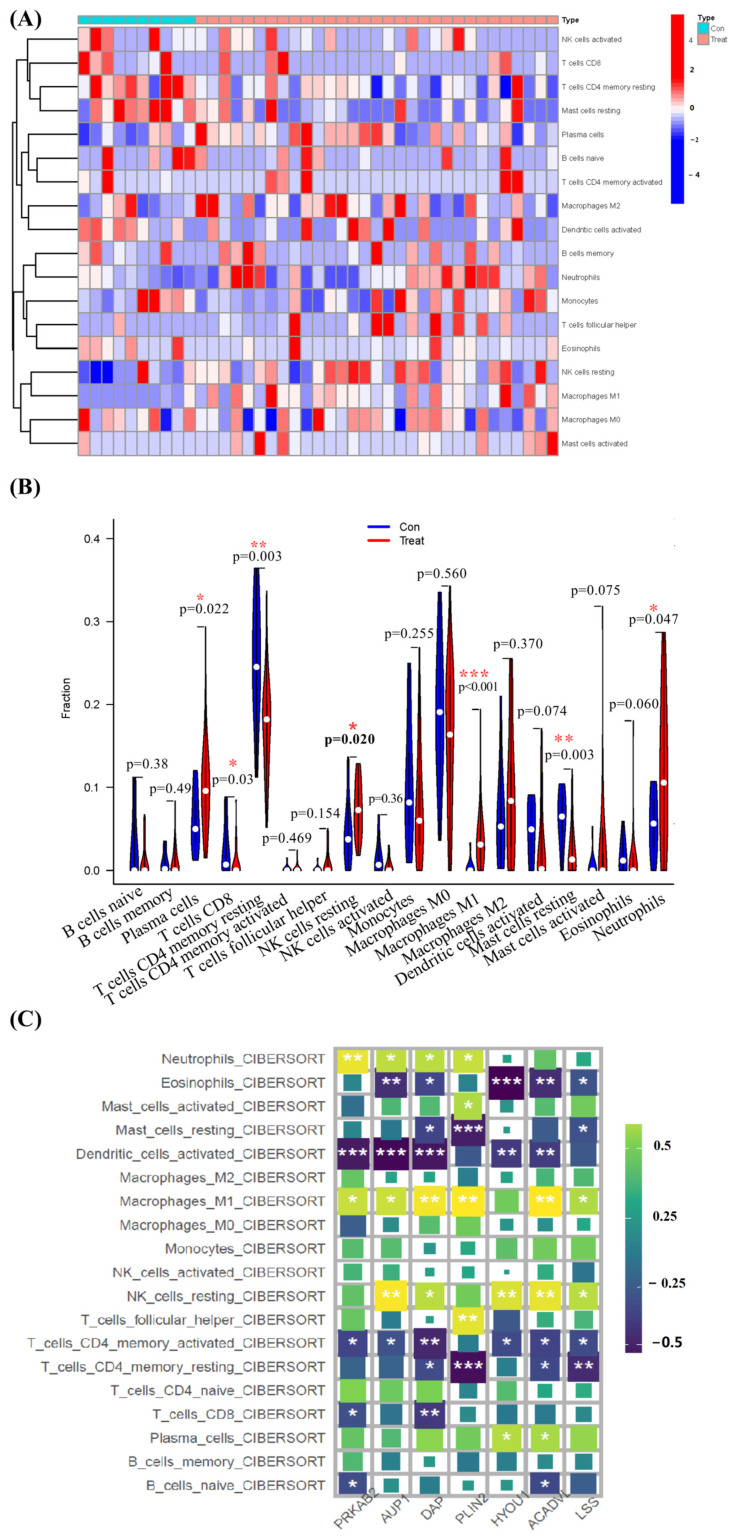
Immune landscape analysis in COVID-19 using the CIBERSORT algorithm. (**A**) The heat plot presenting the proportion of infiltrated immune cells calculated using the CIBERSORT algorithm. (**B**) The differences in immune cells from the immune microenvironment between COVID-19 patients and control samples. (**C**) The correlation diagram of lipophagy biomarkers and immune cells. * *p* < 0.05, ** *p* < 0.01, *** *p* < 0.01, Con (control) vs. Treat (COVID-19) group.

The CIBERSORT algorithm analyzes the enumeration of the 22 immune cell types in 41 samples (GSE183533) (Figure 6, Supplementary Table S1). There were significant differences in the enrichment fraction of plasma cells, T cells CD8, T cells CD4 memory resting, NK cells resting, macrophages M1, mast cells resting, and neutrophils between the two groups (*p* < 0.05~0.001) (Figure 6A). The enrichment fraction of plasma cells, NK cells resting, neutrophils, and macrophage M1 cells in the COVID-19 group were significantly higher than that in the control group (*p* < 0.05~0.001) (Figure 6B). Subsequently, we also explored the relevance of seven diagnostic genes to immune cells. As shown in Figure 6C, Pearson correlation analysis suggested that neutrophils, eosinophils, mast_cells_activated, mast_cells_resting cells, dendritic_cells_activated, macrophages_M1, NK_cells_resting, T_cells_follicular_helper, T_cells_CD4_memory_activated, T_cells_CD4_memory_resting, T_cells_CD8, plasma_cells, and B_cells_naive cells were all associated with seven biomarkers (*ACADVL*, *HYOU1*, *DAP*, *AUP1*, *PRXAB2*, *LSS*, and *PLIN2*). The NK_cells_resting cells, neutrophils, and macrophages_M1 cells were positively correlated with *HYOU1*, *AUP1*, *PRXAB2*, and *PLIN2*, respectively (Supplementary Figure S1). These results suggested that alterations in the immune microenvironment of COVID-19 samples correlated with these seven lipophagy-related biomarkers.

### 3.6. Lipophagy-Related Pathway Analysis

**Figure 7:**
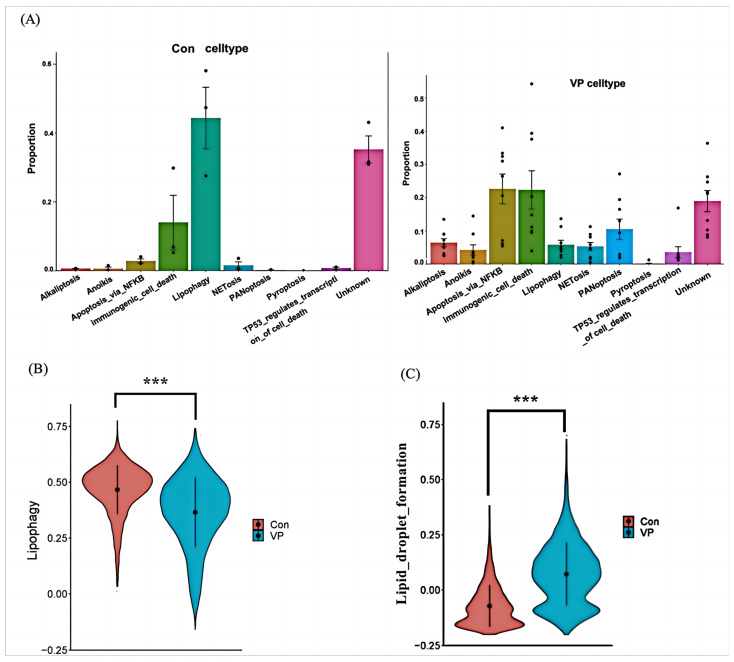
Lipophagy and lipid droplet formation pathway analysis in control and COVID-19 patients from scRNA-seq. (**A**,**B**) Lipophagy pathway. (**C**) Lipid droplet formation pathway. *** *p* < 0.001, Con (control) vs. VP (COVID-19) group.

To further determine the potential role of lipophagy-related signature genes, GSEA pathway analysis was performed. The lipophagy and lipid droplet formation pathways enriched are presented in Figure 7A. The lipophagy pathway was inhibited in COVID-19 patients (Figure 7B). The lipid droplet formation pathway was also activated in COVID-19 patients (Figure 7C). For macrophages, the expression of seven lipophagy-related biomarkers was significantly upregulated in COVID-19 patients (Supplementary Figure S2). Taken together, the results showed that COVID-19 had an increase in the pathway for forming lipid droplets, while the pathway of lipophagy was reduced.

### 3.7. Screening of Drugs Targeting Lipophagy-Related Biomarkers

The cMAP database was utilized to identify the underlying drugs or molecular compounds that could regulate the expression of biomarkers in COVID-19 (Supplementary Table S2 and Table 2). As shown in Table 2, the 11 small molecules or drugs with the highest absolute FC values (|FC| ≥ 0.58) were chosen, which indicated significant correlations with COVID-19. The *AUP1* gene can be inhibited by helveticoside, lanatoside C, and digoxigenin. And lanatoside C, phenoxybenzamine, tretinoin, and tacrolimus can lower *DAP* gene expression. The *HYOU1* gene can be inhibited by geldanamycin, ionomycin, thioridazine, tanespimycin, phenoxybenzamine, diethylstilbestrol, and equilin. Loperamide and trichostatin A can inhibit *LSS* gene expression. *PLIN2* gene expression can be reduced by pioglitazone, rosiglitazone, and troglitazone. Trichostatin A, cephaeline, helveticoside, thioridazine, and lycorine can lower *PRKAB2* gene expression. The *ACADVL* gene can be suppressed by phenoxybenzamine and lanatoside C. It is worth noting that helveticoside can lower the expression of both the *AUP1* and *PRKAB2* genes. Lanatoside C has the ability to suppress the expression of the *AUP1*, *DAP*, and *ACADVL* genes. *DAP*, *HYOU1*, and *ACADVL* gene expression can be reduced by phenoxybenzamine, geldanamycin, tanespimycin, and alvespimycin. Trichostatin A has the ability to suppress the expression of both the *LSS* and PRKAB2L genes. Thioridazine inhibits the expression of both the *HYOU1* and *PRKAB2* genes.

### 3.8. Molecular Docking Analysis Predicts the Binding Modes between Therapeutic Agents and Biomarkers

We used the cMAP database to identify the seven drugs with the best downregulation effect on the seven biomarkers involved in lipophagy. Therefore, molecular docking was used to calculate their binding energies and evaluate their binding scores (Supplementary Table S2). The docking results indicated that the seven drugs were potential inhibitors for each selected target, with moderate to strong binding affinity (−7.5 to −11.8 kcal/mol).

**Figure 8:**
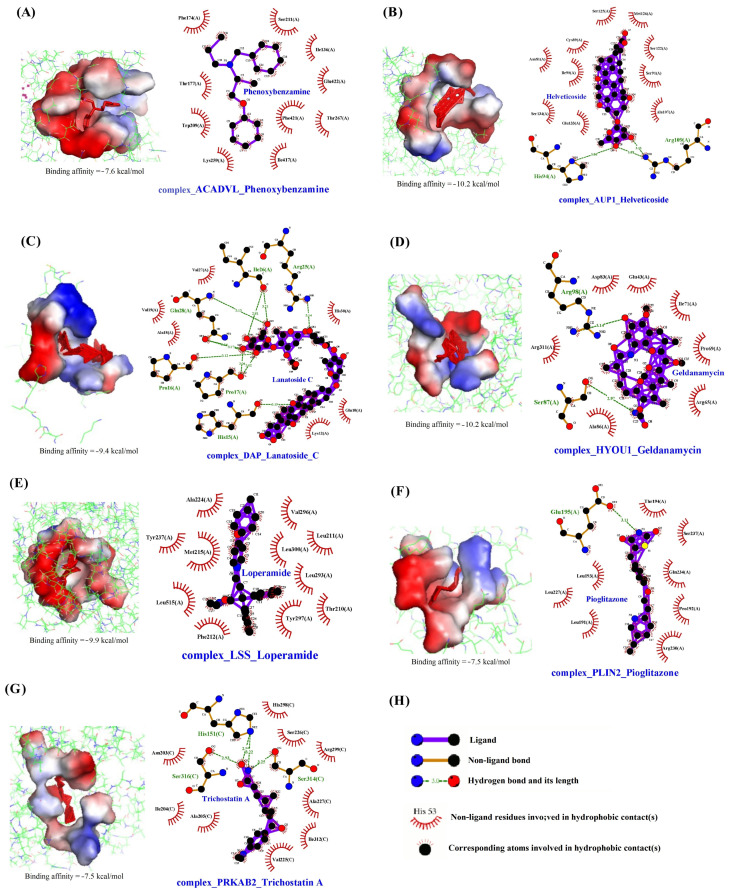
Interaction between seven lipophagy biomarkers and drugs. Interaction of (**A**) ACADVL with phenoxybenzamine, (**B**) AUP1 with helveticoside, (**C**) DAP with lanatoside C, (**D**) HYOU1 with geldanamycin, (**E**) LSS with loperamide, (**F**) PLIN2 with pioglitazone, and (**G**) PRKAB2 with trichostatin A. (**H**) The meaning of the items on the plot. Hydrogen bonds are shown as dotted lines (green).

The best results were obtained for the *ACADVL*-phenoxybenzamine, *AUP1*-helveticoside, *DAP*-lanatoside C, *HYOU1*-geldanamycin, *LSS*-loperamide, *PLIN2*-pioglitazone, and *PRKAB2*-trichostatin A complexes, with free binding energies of −7.6 kcal/mol, −10.2 kcal/mol, −9.4 kcal/mol, −10.2 kcal/mol, −9.9 kcal/mol, −7.5 kcal/mol, and −7.5 kcal/mol, respectively. The seven drugs have good interaction with the seven lipophagy biomarkers. The optimal binding modes of each studied target and drug complex are demonstrated in Figure 8. For *ACADVL*-phenoxybenzamine complexes, hydrophobic actions between phenoxybenzamine and Ser211, Ile136, Glu422, Thr267, Phe421, Ile417, Lys259, Trp209, Thr177, and Phe174 significantly contributed to the stability of the complex (Figure 8A). For *AUP1*-helveticoside complexes, hydrogen bonds between helveticoside and His94 and Arg109, along with hydrophobic contacts between helveticoside and Ser125, Cys89, Asn81, Ile90, Ser134, Glu133, Ala107, Ser91, Ser122, and Met126, significantly contributed to the stability of the complex (Figure 8B). For complex *DAP*–lanatoside C, the hydrogen bonds between lanatoside C with Ile26, Gln28, Pro16, His15, Pro17, and Arg25, and hydrophobic bonds between lanatoside C with Val27, Ala18, Val19, Lys12, Glu10, and His30 further stabilized the structure of the complex (Figure 8C). For *HYOU1*-geldanamycin complexes, hydrogen bonds (Arg98 and Ser87), and hydrophobic contacts (Arg311, Ala86, Arg65, Pro69, Ile71, Glu43, and Asp83) formed between protein residues and geldanamycin contributed to the stability of the complex and were clearly illustrated in Figure 8D. For *LSS*-loperamide complexes, hydrophobic contacts (Ala224, Tyr237, Met215, Leu515, Phe212, Tyr297, Thr210, Leu293, Leu300, Leu211, and Val296) formed between protein residues and loperamide contributed to the stability of the complex and were clearly illustrated in Figure 8E. For *PLIN2*-pioglitazone complexes, hydrogen bonds (Glu195) and hydrophobic contacts (Leu193, Leu227, Leu191, Arg230, Pro192, Gln234, Ser237, and Thr194) formed between protein residues and pioglitazone contributed to the stability of the complex and were clearly illustrated in Figure 8F. For *PRKAB2*-trichostatin A complexes, hydrogen bonds (His151, Ser316, and Ser314) and hydrophobic contacts (Asn203, Ile204, Ala205, Val225, Ile312, Ala227, Arg299, Ser226, and His298) formed between protein residues and trichostatin A contributed to the stability of the complex, as clearly illustrated in Figure 8H.

## 4. Discussion

The continuous evolution of COVID-19 has significantly reduced the effectiveness of current vaccines and drugs. Therefore, it is crucial to identify new targets and biomarkers for the diagnosis and treatment of COVID-19. Viruses, being highly parasitic microorganisms, rely entirely on the metabolic systems of host cells for replication and infection [18], including lipid metabolism [4]. Notably, patients with severe COVID-19 infection have been found to have accumulated lipids in their lungs [8]. Inhibiting key enzymes involved in LD formation has been shown to impact SARS-CoV-2 replication in cells. However, the relationship between COVID-19 and lipid metabolism, particularly lipophagy, remains unclear. 

In this study, we aimed to identify diagnostic biomarkers of lipophagy associated with COVID-19 and investigate the role of lipid metabolism in the disease. We utilized multi-omics data from COVID-19 lung tissues, including 1 scRNAseq and 2 RNAseq datasets, to identify 47 DEGs related to lipophagy between COVID-19 and non-COVID-19 samples. Among these DEGs, 20 genes were upregulated and 27 genes were downregulated. Subsequently, we conducted functional enrichment analysis on these DEGs, revealing their close association with the regulation of autophagy, energy metabolism, and oxidase activity. These findings suggest that COVID-19 is implicated in lipid metabolism, specifically lipophagy and lipid droplet formation, which may contribute to viral infection and inflammation. To identify the key genes associated with lipophagy, we employed four different machine learning algorithms (RF, SVM, GLM, and XGB). The study utilized four machine learning methods (GLM, RF, SVM, and XGB) for training and prediction samples. These methods were employed to sort and select important features for classification. From these models, a total of 40 top important genes associated with lipophagy were identified, including 20 gene repeats and 20 unique genes: *PDIA3*, *FAF2*, *SUCLG1*, *MYH7*, *LDAH*, *PLIN2*, *COPB2*, *PRKAG2*, *P4HB*, *GOT2*, *SCARB2*, *GRPEL1*, *AUP1*, *PRKAB2*, *CS*, *DAP*, *ALDH9A1*, *LSS*, *HYOU1*, and *ACADVL*. Subsequently, seven lipophagy signature genes (*LSS*, *HYOU1*, *ACADVL*, *PRKAB2*, *PLIN2*, *AUP1*, and *DAP*) were identified in the validation set (GSE190496). Gene expression level and ROC curve analysis demonstrated that these seven genes possess good diagnostic ability and may have a significant role in COVID-19. Therefore, they were recognized as biomarkers and potential therapeutic targets for COVID-19.

We conducted an analysis of BALF using single-cell RNA sequencing data from patients with COVID-19 disease and healthy subjects. Our findings revealed the presence of various cell types, including stem-like cells, airport goblet cells, chemotaxis, ciliated cells, interstitial macrophages, macrophages, neutrophils, and plasma cells, in COVID-19 patients. The number of immune cells, particularly macrophages, showed a significant increase. Additionally, we observed that lipid droplets, which store neutral lipids such as triglycerides and cholesterol, are multifunctional organelles involved in various cellular processes. These droplets also have a close association with the infection and pathogenesis of pathogenic microorganisms. 

Pathogenic microorganism infection not only alters the size and quantity of lipid droplets in host cells but also facilitates the proliferation of pathogens by utilizing lipid droplets as platforms for assembly or replication. Various pathogens, such as HCV, EV71, and DENV, specifically target and interact with lipid droplets. Inhibiting this binding or suppressing lipid droplet generation can result in a reduction in virus replication ability. Viral infections, such as hepatitis C and dengue, induce the formation of lipid droplets in host cells. Additionally, inhibiting the biosynthesis of lipid droplets using drugs can significantly impede the replication of DENV and HCV [19]. In patients who succumbed to COVID-19, the lung tissue displayed DMVs filled with viral RNA alongside the presence of LD in close proximity to DMVs [8]. Our investigation revealed an upregulation of the lipid droplet synthesis pathway in the BALF of COVID-19 patients. Pharmacological inhibition of key enzymes involved in lipid droplet formation affects the replication of SARS-CoV-2 in cells [20]. These findings suggest that targeting lipid accumulation could be a promising approach for combating COVID-19.

Lipophagy is an intracellular pathway responsible for the degradation of fat. It involves the transportation of lipid droplets and cholesterol within the cell through autophagosomes to lysosomes. These lipid droplets are then broken down into glycerol and free fatty acids by acid lipase and hydrolase enzymes after fusion [5]. Lipophagy plays a crucial role in maintaining the balance of intracellular lipid metabolism and also creates favorable conditions for the infection and replication of pathogenic microorganisms [6]. Inhibition of lipophagy can result in excessive accumulation of lipids and lead to tissue damage and inflammation-related diseases. Various pathogenic microorganisms, such as SARS-CoV-2, HBV, HCV, and dengue virus, have been found to inhibit lipophagy, thereby attenuating immune responses [9]. The excessive accumulation of LDs in the various organs of obese individuals can contribute to the replication of SARS-CoV-2 and hinder its elimination. This is due to mechanisms associated with lipid overload [10]. Our study observed a downregulation of lipophagy and an upregulation of the TP53-regulated cell death pathway in the BALF of COVID-19 patients. Therefore, the use of drugs or natural products that stimulate LD clearance by promoting lipid autophagy could potentially reduce virus replication and enhance its elimination through viral phages. This approach holds significant promise for the treatment of COVID-19.

To comprehensively study the role of immune cells in COVID-19, we utilized the CIBERSORT algorithm in this study to assess the immune infiltration status. We observed an increase in the abundance of plasma cells, NK cell resting, neutrophils, and macrophage M1 cells. These cells are crucial components of the body’s adaptive immunity and may play a significant role in the pathogenesis of COVID-19 [21]. Additionally, we found a significant correlation between seven characteristic genes of lipophagy and different immune cells. The expression of these genes on immune cells was also positively correlated, suggesting their potential involvement in autoimmunity. It is necessary to further study the molecular mechanism and function of immune cell infiltration in COVID-19 cells.

This study utilized the cMAP database to identify seven lipophagy signature genes and explore therapeutic agents for combating COVID-19. Computer docking studies revealed that these seven drugs demonstrated promising potential in inhibiting the effects of *ACADVL*, *HYOU1*, *DAP*, *AUP1*, *PRXAB2*, *LSS*, and *PLIN2*. These genes are all involved in lipid metabolism, which plays a crucial role in various stages of the viral infection replication cycle, including viral adsorption, viral nucleic acid entry into the nucleus, viral genome replication and transcription, viral encoded protein translation, virion assembly, and the release of progeny virions. Abnormal lipid metabolism is a key feature in various viral infections. Impaired lipid metabolism is linked to inflammation and cell death [22]. Disruptions in lipid metabolism often result in the accumulation of cellular ROS and the release of inflammatory factors, which contribute to inflammatory pathological reactions [23]. Notably, the inflammatory pathological response is a significant characteristic of COVID-19 infection. The coronavirus has the ability to induce high membrane plasticity in host cells, and its nonstructural proteins facilitate the formation of DMVs [24].

*ACADVL* (acyl CoA dehydrogenase very long chain) is a catalytic mitochondrial enzyme involved in the oxidation of fatty acids. This process occurs in the presence of oxygen and results in the breakdown of fatty acids into acetyl CoA, which is then used for energy production from fat [25]. The expression of *ACADVL* promotes fatty acid β-oxidation. In the context of COVID-19 infection, it has been observed that the virus can manipulate the host cell’s fatty acid oxidation (FAO) to create a favorable environment for its replication. The increased expression of *ACADVL* during COVID-19 infection suggests that SARS-CoV-2 may exploit *ACADVL* to promote lipid metabolism and facilitate virus transmission. Phenoxybenzamine, an alpha-adrenergic blocker, has been utilized for the treatment of hypertension and as a peripheral vasodilator. It acts by dilating peripheral blood vessels and improving microcirculation [25]. Additionally, phenoxybenzamine functions as a calmodulin antagonist, inhibiting the binding of the glucocorticoid receptor heat shock protein-90 complex to hormones. Hsp90 is crucial for the replication of various viruses, such as HBV, HCV, HCMV, HSV, rhinovirus, and Ebola virus [26]. Certain viruses rely on cellular Hsp90 for the folding and assembly of viral structural proteins, as well as for the maturation of viral enzymes. Hsp90 also plays a significant role in the life cycle of SARS-CoV-2. EIPs (ATP6V0C or vATPase) are vital for viral entry and are involved in cell proliferation and differentiation [27]. In this study, it was discovered that phenoxybenzamine downregulated the *ACADVL* genes, potentially inhibiting the replication of SARS-CoV-2 by regulating lipid metabolism imbalance.

The protein encoded by *AUP1* (ancient ubiquitous protein 1) primarily functions in lipid storage and lipid droplet accumulation [28]. *AUP1* controls lipid synthesis by inducing ubiquitination and the subsequent degradation of several key regulators of lipid biosynthesis (e.g., 3-hydroxy-3-methylglutaryl-CoA reductase (HMGCR) and ubiquitin conjugating enzyme E2 G2 (UBE2G2)). Therefore, the expression of *AUP1* affects the number and size of LDs and plays a crucial role in LD regulation [29]. Virus-triggered lipophagy is essential for virus assembly and is driven by the lipid droplet-associated protein *AUP1*. *AUP1* is highly expressed during COVID-19 infection, indicating its significance in the accumulation of lipid droplets triggered by the SARS-CoV-2 virus and the assembly and secretion of viral progeny. Helveticoside is a biologically active component found in the seed extract of *Descurainia sophia* [30]. In a COVID-19 patient, helveticoside has been found to downregulate *UBE2C* expression, leading to the inhibition of viral proliferation and apoptosis [31]. This study also revealed that helveticoside has a downregulation effect on *AUP1*, which can inhibit the accumulation of lipid droplets caused by the virus. Consequently, helveticoside exhibits antiviral effects by inhibiting virus proliferation, differentiation, and apoptosis.

*DAP* (death-associated protein) encodes a proline-rich 15 KD basic protein [32]. The *DAP* gene is involved in apoptosis and plays a role in mediating interferon γ-induced cell death. *DAP* is a substrate of mTOR and acts as a negative regulator of autophagy [33]. In this study, it was found that *DAP* was highly expressed during COVID-19 infection, suggesting that SARS-CoV-2 manipulated the *DAP* gene to reduce lipophagy, thereby accelerating virus replication and progression. Lanatoside C, a cardiac glycoside, can block the binding between host ACE2 and the S protein of SARS-CoV-2, preventing the virus from entering the target cells. Lanatoside C also downregulates *UBE2C* and exhibits antiviral effects [31]. In this study, it was observed that lanatoside C downregulated *DAP*, promoted lipophagy, and demonstrated antiviral activity.

*HYOU1* (hypoxia upregulated 1) is an ER resident chaperone and a member of the heat shock and ER stress protein family [34]. It is expressed in various cell types and can be upregulated by different cellular conditions, including hypoxia and ER stress. SARS-CoV-2 infection can produce many pathophysiological changes, such as inflammation and immune response dysregulation, oxidative stress, hypercoagulable state, capillary damage, and tissue hypoxia [35], as well as instability in glycemic control, which leads to an increased expression level of *HYOU1*. Hsp90, another cellular chaperone, has a unique role in inducing the post-translational maturation of specific transcription factors, kinases, and steroid hormone receptors. It also plays an important role in the SARS-CoV-2 life cycle [31]. Geldanamycin, an Hsp90 inhibitor, has been found to inhibit the replication of various viruses by suppressing the activity of Hsp90. Moreover, geldanamycin impedes the normal functioning of viral proteins by preventing the formation of complexes that involve Hsp90 and viral proteins [36]. A cytokine storm, which is associated with the activation of proinflammatory mediators such as nuclear factors κB (NF-κB) and mitogen-activated protein (MAP) kinase, requires the stabilization and functioning of IKB kinase (IKK) complexed with Hsp90 [37]. Geldanamycin has also been observed to downregulate the expression of the cell cycle genes CCNB1 and UBE2C in COVID-19 [31], which can potentially serve as effective antiviral and anti-inflammatory agents [38]. In our study, geldanamycin was found to downregulate the expression of *HYOU1*, inhibit lipid peroxidation, regulate lipid metabolism disorders, and exhibit antiviral, antioxidant, and anti-inflammatory activities.

*LSS* (lanosterol synthase) is a crucial enzyme in the cholesterol biosynthesis pathway. It primarily catalyzes the conversion of (s)-2,3 oxidosqualene to lanosterol, which is a key step in limiting the rate of cholesterol biosynthesis [39]. The impact of other components of the cholesterol biosynthesis pathway on viral pathogen replication has been studied [40]. Inhibition of *LSS* can enhance the antiviral IFN-β response induced by the respiratory syncytial virus (RSV). Loperamide, a phenylpiperidine opioid, is commonly used to treat diarrhea. Diarrhea affects approximately 10% to 20% of COVID-19 patients. Studies have demonstrated that loperamide can induce cell death dependent on Atg5 and Atg7, which is associated with an increase in proteins related to lipid and cholesterol metabolic processes [41]. In vitro experiments have shown that loperamide can inhibit the replication of SARS coronavirus and human coronavirus 229E [42]. In this study, it was found that loperamide can downregulate the expression of *LSS* in patients with SARS-CoV-2. This downregulation inhibits the synthesis of cholesterol and cellular lipophagy, which in turn hinders the proliferation and differentiation of the virus. 

The protein encoded by *PLIN2* (perilipin 2) is responsible for covering intracellular lipid storage droplets and plays a role in the development and maintenance of adipose tissue. It serves as a marker for lipid accumulation in various cell types and diseases [43]. *PLIN2* overexpression inhibits autophagy, while *PLIN2* deficiency promotes autophagy [44]. Additionally, *PLIN2* regulates genes involved in metabolic functions, including lipid metabolism- and inflammatory response-related genes [45]. In this study, we observed that *PLIN2* expression levels were upregulated in the BALF of COVID-19 patients and the lungs of deceased patients. Pioglitazone, a peroxisome proliferator-activated receptor γ (PPAR γ) agonist, is commonly used as an insulin sensitizer in clinical practice. Human studies have shown that pioglitazone can enhance cytosolic lipolysis, β-oxidation, and autophagy, leading to the improvement of hepatic steatosis [46]. PPARs are a group of transcription factors involved in insulin response, regulating glycemic control, lipogenesis, and inflammation. Pioglitazone exhibits various anti-inflammatory activities, including the significant reduction of IL-6 and tumor necrosis factor α in individuals with insulin resistance [47]. Therefore, pioglitazone may potentially be used to reduce COVID-19-related inflammation and the associated risk of death. Our findings suggest that pioglitazone can downregulate *PLIN2* expression, indicating that its antiviral effect may be partly mediated by alterations in cellular lipids.

*PRKAB2* (protein kinase AMP-activated non-catalytic subunit beta 2) encodes the regulatory subunit of AMP-activated protein kinase (AMPK). AMPK is composed of α catalytic subunits and non-catalytic β and γ subunits, which form heterotrimers. These heterotrimers phosphorylate and inactivate acetyl CoA carboxylase (ACC) and hydroxymethylglutaryl CoA reductase (HMGCR), key enzymes involved in regulating the de novo biosynthesis of fatty acids and cholesterol [48]. The AMPK pathway has been reported to regulate the expression of PPAR-γ and C/EBPα, further contributing to the inhibition of preadipocyte to adipocyte differentiation [49]. This study discovered that in patients with COVID-19, PRKAB2 was upregulated, suggesting that SARS-CoV-2 manipulated the PRKAB2 gene, leading to enhanced lipid metabolism and accelerated viral replication and differentiation. Trichostatin A (TSA), a histone deacetylase (HDAC) inhibitor, has various pharmacological functions, including anti-inflammatory and antitumor properties and neuroprotection [50]. TSA has been shown to inhibit pro-inflammatory cytokines (e.g., IL-6 and IL-8), cell growth, and the proliferation of virus-infected cells [51]. In this study, it was observed that TSA could downregulate the expression of PRKAB2, suggesting that its antiviral activity might be partly attributed to its regulation of lipid metabolism, anti-inflammatory, antioxidant, and other effects.

In sum, this study identifies seven lipophagy-related genes (*ACADVL*, *HYOU1*, *DAP*, AUP1, *PRXAB2*, *LSS*, and PLIN2) as biomarkers and potential therapeutic targets for COVID-19. Additionally, phenoxybenzamine, helveticoside, lanatoside C, geldanamycin, loperamide, pioglitazone, and trichostatin A show promise as therapeutic agents for COVID-19. However, it is important to note that this study has limitations. The size of the test as well as the validation data set were limited. As it was based on retrospective analysis, further research, validation, and in vivo, in vitro, and clinical studies are necessary to fully understand the functions of these key genes and the effectiveness of the predicted therapeutic agents. Nevertheless, the study provides valuable insights into the potential drug action and mechanism of targeting lipophagy-related genes to treat COVID-19. It can serve as a useful reference for understanding the impact of such interventions.

## 5. Conclusions

The lipophagy-related genes *ACADVL*, *HYOU1*, *DAP*, *AUP1*, *PRXAB2*, *LSS*, and *PLIN2* can be used as biomarkers and drug targets for COVID-19. Seven potential downregulators of the seven biomarkers may have therapeutic effects for COVID-19.

## Figures and Tables

**Table 1:** Lipophagy-related DEGs between COVID-19 samples and control samples.

Gene	logFC	AveExpr	*p*-Value	Type
ACADVL	1.22056	12.37652	7.12 × 10^−6^	Up
ANXA2	−1.04974	12.14506	8.36 × 10^−5^	Down
ARHGDIB	−1.22168	9.722035	0.001734	Down
AUP1	0.73916	9.827391	0.000167	Up
CHMP4B	−1.0309	9.196918	4.33 × 10^−5^	Down
COPB2	0.625736	10.93317	1.97 × 10^−5^	Up
CORO1A	−1.47724	8.119408	0.020948	Down
CS	0.832411	10.24263	1.27 × 10^−7^	Up
CTSS	−1.82733	10.72099	3.45 × 10^−11^	Down
DAP	1.040417	9.901216	2.28 × 10^−6^	Up
FABP4	−4.77581	6.214894	2.87 × 10^−12^	Down
GOT2	2.209275	9.779301	6.45 × 10^−9^	Up
GRPEL1	1.30356	8.682127	4.15 × 10^−6^	Up
HSP90AA1	−1.08199	12.62869	0.004237	Down
HSPA9	1.026774	11.79201	5.30 × 10^−5^	Up
HSPB1	−1.44516	9.321457	0.001028	Down
HYOU1	0.712801	11.31537	0.002449	Up
LAMTOR5	−1.02744	7.687586	1.60 × 10^−5^	Down
LDAH	0.73637	7.179144	0.013203	Down
LSS	0.806331	10.79817	0.011611	Up
MAN2B1	−0.7043	9.15311	0.017938	Down
MDH2	1.148977	10.18082	7.00 × 10^−5^	Up
MYH7	5.128091	6.764768	5.66 × 10^−5^	Up
MYH9	−0.84748	13.85285	0.006738	Down
MYL12B	−0.50209	10.53187	0.001045	Down
MYL6	−1.43376	11.30935	3.41 × 10^−12^	Down
NPC2	−1.78017	9.491113	3.29 × 10^−6^	Down
P4HB	1.488593	12.91802	8.95 × 10^−8^	Up
PARK7	−0.73148	9.06782	3.65 × 10^−7^	Down
PDIA3	0.656054	11.55285	0.002806	Up
PDIA4	0.916355	11.03247	0.000118	Up
PLIN2	1.762544	11.95768	6.69 × 10^−6^	Up
PRKAB2	1.463509	9.281841	1.60 × 10^−5^	Up
PRKAG2	1.206049	9.86256	1.74 × 10^−6^	Up
PSAP	−0.55917	13.51833	0.018188	Down
RAB11B	−0.56735	8.530345	0.026198	Down
RAB6B	−1.77904	4.64767	0.000305	Down
RHOA	−0.58227	11.71363	0.000584	Down
RPS27A	−1.40497	10.60716	2.02 × 10^−8^	Down
SCARB2	0.639907	11.73706	0.000243	Up
SUCLG1	0.637036	9.091021	0.005076	Up
UBB	−1.08032	11.27086	4.96 × 10^−5^	Down
UBC	−0.63646	13.17368	0.020104	Down
VIM	−1.67728	12.38277	0.000149	Down
YWHAE	−0.61307	10.97161	7.56 × 10^−5^	Down
YWHAQ	−0.67158	10.06487	5.48 × 10^−5^	Down

**Table 2:** Drugs with down-regulation mRNA expression of seven feature genes in cMAP database (|Fold Change| > 0.58).

Drug Name	Gene Name	Molecular Formula	Fold Change	Description
*Helveticoside*	AUP1	C_29_H_42_O_9_	−0.925626	Helveticoside is a cardenolide glycoside.
Lanatoside C		C_49_H_76_O_20_	−0.820001	Lanatoside C is a cardiac glycoside with antiviral and antitumor activities
Digoxigenin		C_23_H_34_O_5_	−0.719413	A toxic cardiac glycoside mainly from digitalis.
*Lanatoside C*	DAP	ditto	−0.78183	ditto
Phenoxybenzamine		C_18_H_22_ClNO	−0.62779	An alpha-adrenergic antagonist with long duration of action.
Tretinoin		C_20_H_28_O_2_	−0.6105	All-trans-retinoic acid (ATRA), is a naturally occurring derivative of vitamin A (retinol).
Tacrolimus		C_44_H_69_NO_12_	−0.60699	Tacrolimusis an immunosuppressive drug and chemically known as a macrolide.
*Geldanamycin*	HYOU1	C_29_H_40_N_2_O_9_	−1.54383	Geldanamycin is a benzoquinone antineoplastic antibiotic.
Ionomycin		C_41_H_72_O_9_	−1.28528	Ionomycin is a polyether antibiotic isolated from Streptomyces conglobatus sp. nov. Trejo with antineoplastic activity.
Thioridazine		C_21_H_26_N_2_S_2_	−0.97859	A phenothiazine antipsychotic used in the management of psychoses
Tanespimycin		C_31_H_43_N_3_O_8_	−0.95051	Tanespimycin is a benzoquinone antineoplastic antibiotic.
Phenoxybenzamine		ditto	−0.85209	ditto
Diethylstilbestrol		C_18_H_20_O_2_	−0.85105	Diethylstilbestrol is an olefinic compound. An antineoplastic agent, a carcinogenic agent, an autophagy inducer and a calcium channel blocker.
Equilin		C_18_H_20_O_2_	−0.66944	Equilin is a naturally occurring steroid with estrogenic activity.
*Loperamide*	LSS	C_29_H_33_ClN_2_O_2_	−0.77261	Loperamide is an anti-diarrheal agent that is structurally similar to opiate receptor agonists such as diphenoxylate and haloperidol.
Trichostatin A		C_17_H_22_N_2_O_3_	−0.74973	Trichostatin A is an antibiotic antifungal agent, a trichostatin and a hydroxamic acid.
*Pioglitazone*	PLIN2	C_19_H_20_N_2_O_3_S	−0.759247	Pioglitazone is a Peroxisome Proliferator Receptor alpha Agonist, and Peroxisome Proliferator Receptor gamma Agonist, and Thiazolidinedione.
Rosiglitazone		C_18_H_19_N_3_O_3_S	−0.726808	Rosiglitazone is an anti-diabetic drug and selective ligand of PPARγ.
Troglitazone		C_24_H_27_NO_5_S	−0.671461	Troglitazone has a role as a hypoglycemic agent, an antioxidant, a vasodilator agent, an anticonvulsant, an anticoagulant, a platelet aggregation inhibitor, an antineoplastic agent, and a ferroptosis inhibitor.
*Trichostatin A*	PRKAB2	ditto	−1.07525	ditto
Cephaeline		C_28_H_38_N_2_O_4_	−0.79435	Cephaeline is a phenolic alkaloid in Indian ipecac. Cephaeline has high inhibitory effect on virus zikv and EBOV infection.
Helveticoside		ditto	−0.75729	ditto
Thioridazine		ditto	−0.69689	ditto
Lycorine		C_16_H_17_NO_4_	−0.61567	Lycorine is an indolizidine alkaloid. It has a role as a protein synthesis inhibitor, an antimalarial, a plant metabolite and an anticoronaviral agent.
*Phenoxybenzamine*	ACADVL	ditto	−0.58961	ditto
Lanatoside C		ditto	−0.58311	ditto

## Data Availability

All data generated or analyzed during this study are included in this article/Supplementary Materials.

## Supplementary Materials

The following supporting information can be downloaded at: https://www.mdpi.com/article/10.3390/v16060923/s1, Figure S1: The correlation between lipophagy feature genes and macrophages M1 cells. (A) ACADVL; (B) HYOU1; (C) DAP; (D) AUP1; (E) PRXAB2; (F) LSS; (G) PLIN2; Figure S2: Expression of seven lipophagy related feature genes in macrophage and correlation to lipid droplet formation score. (A) ACADVL; (B) AUP1; (C) DAP; (D) HYOU1; (E) LSS; (F) PLIN2; (G) PRKAB2. Table S1: The cell enumeration level of CIBERSORT in COVID-19; Table S2: The screened results of the possible compounds using cMAP database.

## Abbreviations

COVID-19, coronavirus disease 2019; LD, lipid droplets; GO, gene ontology; KEGG, Kyoto Encyclopedia of Genes and Genomes; DEGs, differentially expressed genes; ROC, receiver operating characteristic; ANOVA, analysis of variance; AUC, area under curve; FC, fold change; BP, biological process; CC, cellular composition; MF, molecular function; HBV, hepatitis B virus; HCV, hepatitis B virus; ATP, adenosine triphosphate; DMVs, double-membrane vesicles; scRNA-Seq, single-cell RNA sequencing; SARS-CoV-2, severe acute respiratory syndrome coronavirus 2; GEO, gene expression omnibus; BALF, bronchoalveolar lavage fluid; UMAP, uniform manifold approximation and projection; FDR, false discovery rate; SVM, support vector machine; RF, random forest; XGBoost, extreme gradient boosting; GLM, generalized linear model; GSVA, gene set variation analysis; ACADVL, acyl CoA dehydrogenase very long chain; AUP1, ancient ubiquitous protein 1; ER, endoplasmic reticulum; TGEV, transmissible gastroenteritis virus; DAP, death-associated protein; HYOU1, hypoxia upregulated 1; IKK, IKB kinase; NF-κB, nuclear factors κB; MAP, mitogen-activated protein; LSS, lanosterol synthase; PLIN2, perilipin 2; PPAR, peroxisome proliferator-activated receptor; PRKAB2, protein kinase AMP-activated non-catalytic subunit beta 2; AMPK, AMP-activated protein kinase; ACC, acetyl CoA carboxylase; HMGCR, hydroxymethylglutaryl CoA reductase; TSA, trichostatin A
