# Supplementary material for: Identification and Analysis of Biomarkers Associated with Lipophagy and Therapeutic Agents for COVID-19
Source: Viruses. 2024 Jun 7;16(6):923. doi: 10.3390/v16060923 (PMC11209609; doi:10.3390/v16060923)
Supplement: Supplementary file 1 [file viruses-16-00923-s001.zip › Supplementary Figures S1-S2.pdf]

Supplementary Material

Identification and Analysis of Biomarkers Associated with Lipophagy and Therapeutic Agents for COVID-19

FIGURES

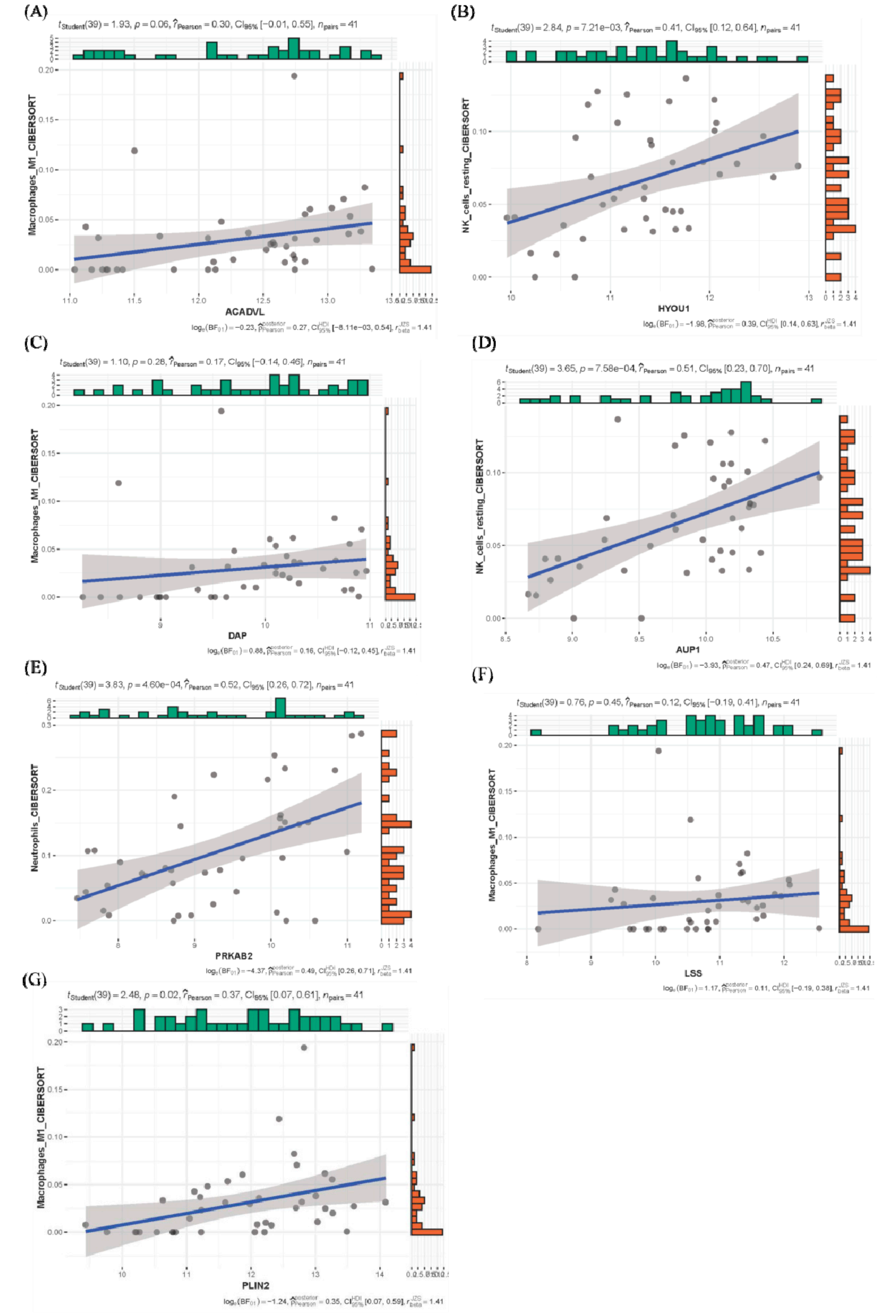

Figure S1. The correlation between lipophagy feature genes and macrophages M1 cells. (A)ACADVL; (B)HYOU1; (C)DAP; (D)AUP1; (E)PRXAB2; (F)LSS; (G)PLIN2.

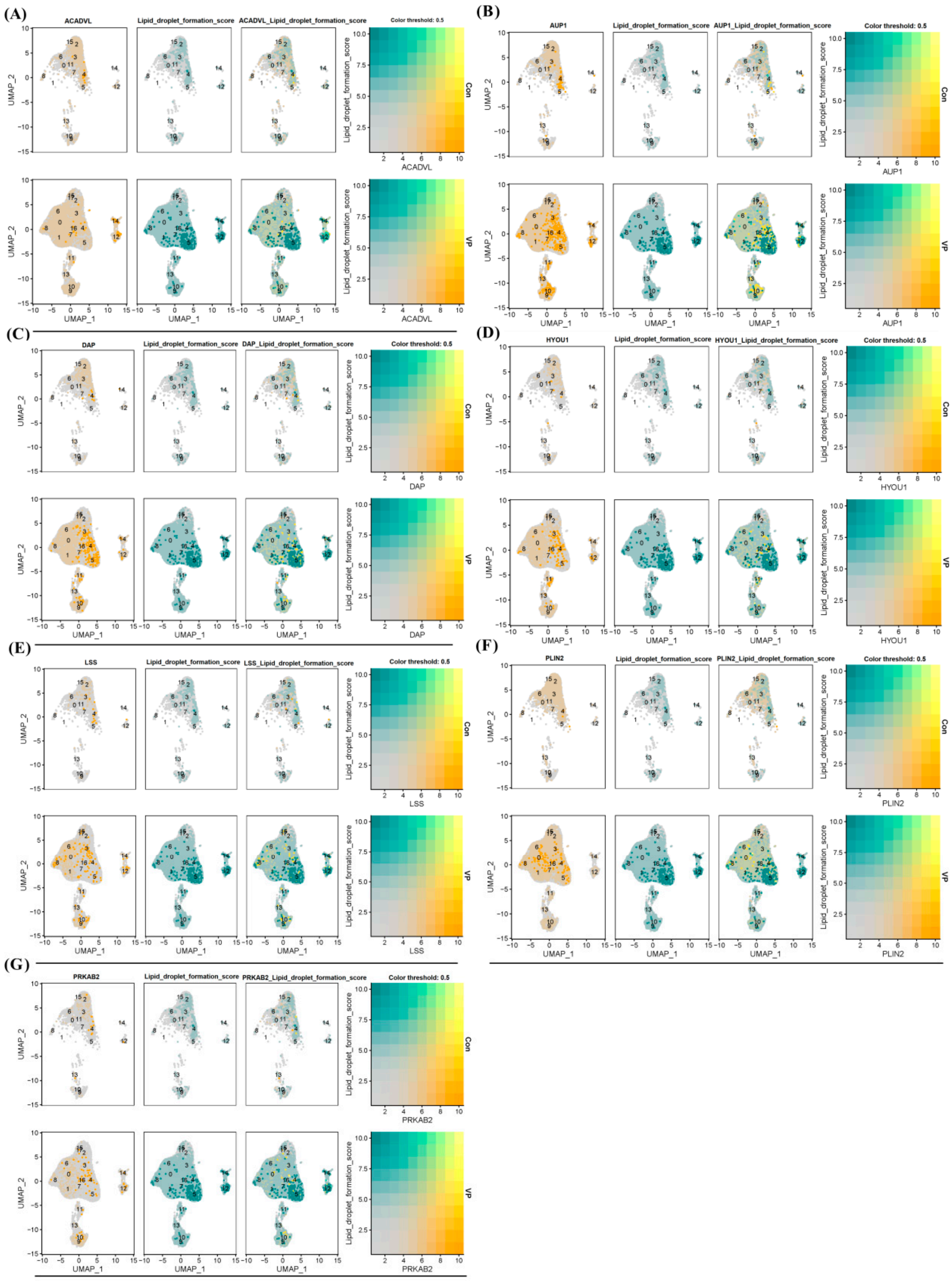

Figure S2 Expression of seven lipophagy related feature genes in macrophage and correlation to lipid droplet formation score. (A) ACADVL;(B) AUP1;(C) DAP;(D) HYOU1;(E)LSS;(F) PLIN2;(G)PRKAB2.
